# Utilization of Point-of-Care Ultrasound to Detect Splenic Microabscesses in Pediatric Melioidosis

**DOI:** 10.7759/cureus.13760

**Published:** 2021-03-08

**Authors:** Teeranai Sakulchit, Louise Ngu, Yek Kee Chor, Gene Y Ong

**Affiliations:** 1 Department of Emergency Medicine, Songklanagarind Hospital, Prince of Songkla University, Hatyai, THA; 2 Department of Pediatrics, Sarawak General Hospital, Sarawak, MYS; 3 Children’s Emergency, KK Women’s and Children’s Hospital, Singapore, SGP

**Keywords:** point of care ultrasound, splenic abscess, melioidosis, children

## Abstract

Melioidosis is an infectious disease most commonly found in places with tropical climates. Definitive diagnosis can be confirmed by culture or pathological results of blood or infected organ. However, imaging study is helpful in providing early provisional diagnosis and guiding therapy. Point-of-care ultrasound can be currently performed bedside by non-radiological staff such as emergency physicians or intensivists. We present the case of a pediatric patient who got diagnosed with melioidosis after detection of multiple splenic and hepatic abscesses by point-of-care ultrasound, leading to early diagnosis and appropriate empirical antibiotic selection, resulting in good treatment outcome.

## Introduction

Melioidosis is an infectious disease caused by the bacterium *Burkholderia pseudomallei*, which is a bipolar-staining Gram-negative aerobic bacillus most commonly found in the soil and water of places with tropical climates, especially South East Asia and Northern Australia [1]. The incidence of melioidosis in Singapore is 1.7 in 100,000 people compared with other countries in the same region like Thailand, which has an incidence of 4.4 in 100,000 people [2], and central Sarawak of Malaysia, which has an incidence of 4.1 in 100,000 children less than 15 years of age [3]. Those with occupational exposure to soil and those with chronic diseases especially diabetes mellitus have a higher risk of infection [1,4,5]. Clinical manifestations include fever, respiratory (e.g., cough or respiratory distress), and abdominal symptoms (e.g., abdominal pain or discomfort). Definitive diagnosis is confirmed by positive blood, aspirates, or tissue cultures. Serology testing by indirect hemagglutination (IHA) with high titer (>1:640) or four-fold rising may also contribute to the diagnosis [2]. However, imaging studies such as ultrasound, CT, or MRI are also helpful in the diagnosis [1].

We report the case of a child with persistent fever who visited our institute in Malaysia. Point-of-care ultrasound (POCUS) of the abdomen was performed, which revealed multiple hepatic and splenic abscesses, leading to the diagnosis of melioidosis. The consent for publishing the child’s medical information was obtained from the caregiver.

## Case presentation

An eight-year-old previously healthy Asian boy presented with fever of 38.5 degree Celsius for 13 days with cough, diarrhea, and limping due to ankle pain. On physical examination, he was lethargic, was in severe respiratory distress, and had a distended abdomen and hepatosplenomegaly. He was noted to be in severe septic shock with impending cardiorespiratory failure. He was emergently intubated and fluid resuscitated, and required multiple vasoactive agents for hemodynamic stabilization. Post-initial stabilization, he was transferred to the intensive care unit (ICU) for further care. His full blood count revealed a total white blood cell count of 4.2 x 10^3^/uL, platelet of 179 x 10^3^/uL, and hemoglobin of 8.9 g/dL. Liver function test showed total bilirubin of 15 umol/L, direct bilirubin of 13 umol/L, aspartate transaminase (AST) of 735 U/L, alanine transaminase (ALT) of 180 U/L, alkaline phosphatase (ALP) of 213 U/L, total protein of 63 g/L, albumin of 22 g/L, and globulin of 40 g/L. His coagulation tests revealed prothrombin time (PT) of 18.8 seconds, international normalized ratio (INR) of 1.84, and activated partial thromboplastin time (aPTT) of 38.5 seconds.

**Figure 1:**
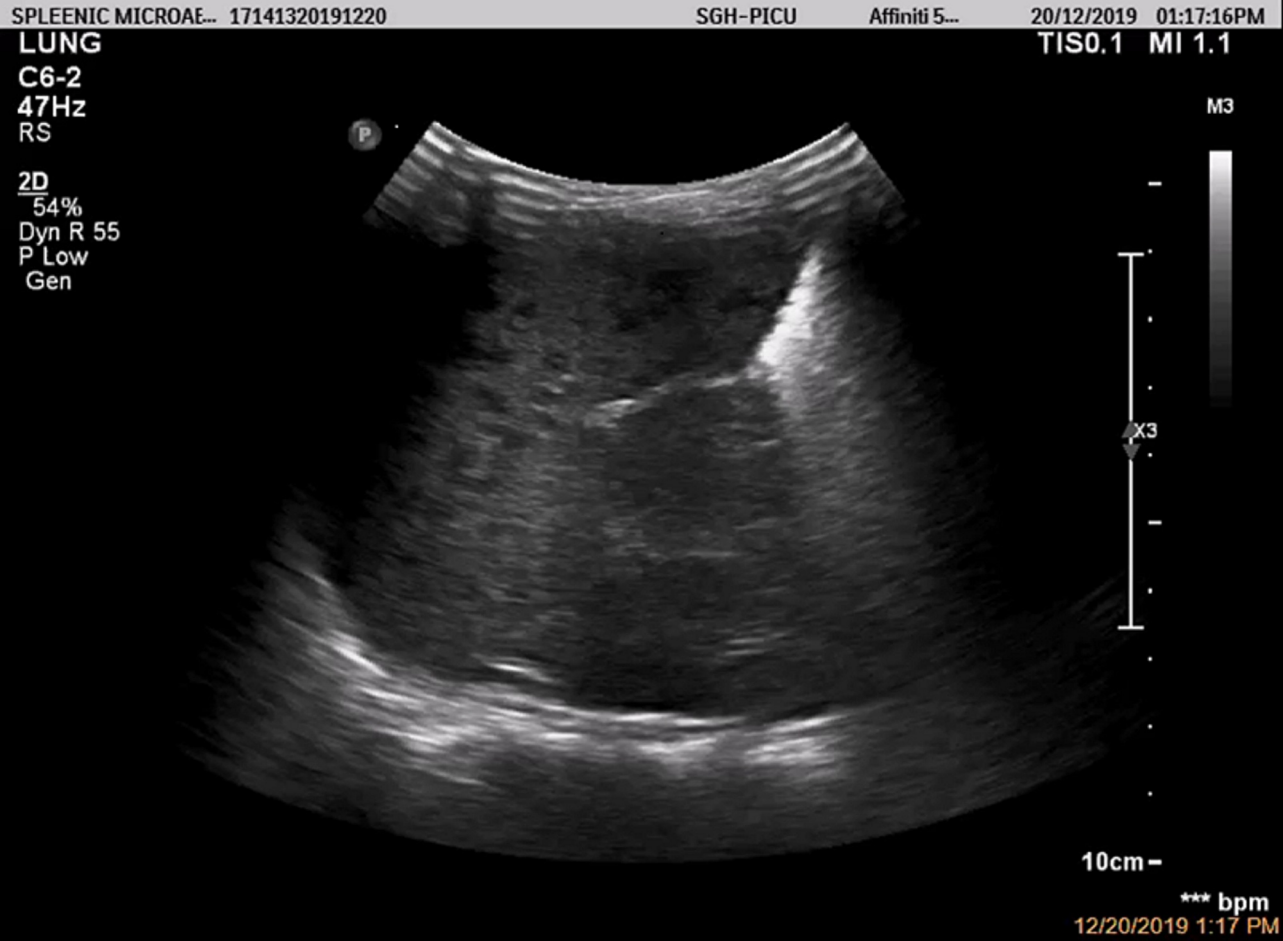
Multiple splenic abscesses seen on the point-of-care ultrasound using a curvilinear transducer.

**Figure 2:**
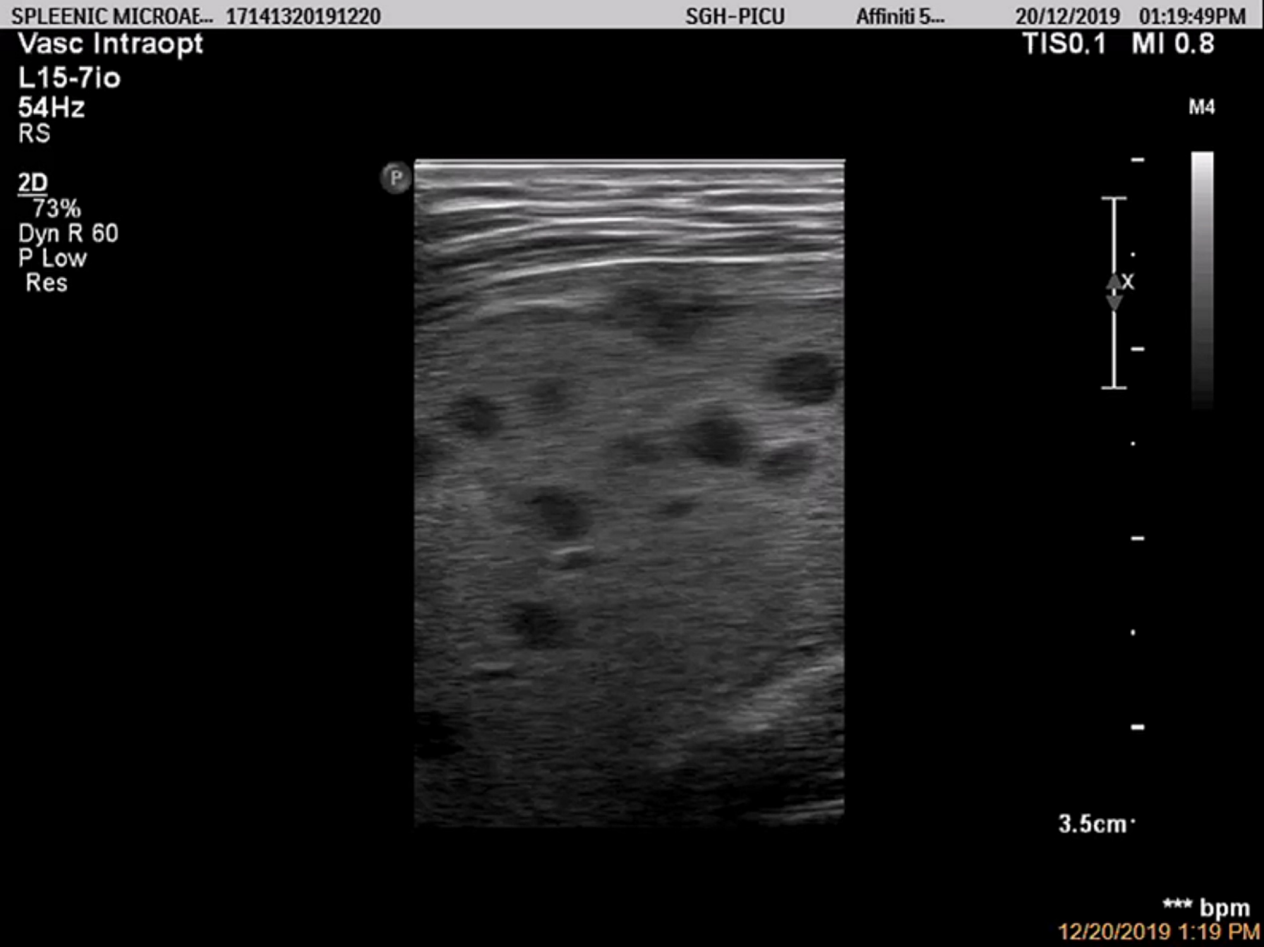
Multiple splenic abscesses seen on the point-of-care ultrasound using a linear transducer.

POCUS of the abdomen was therefore performed by a pediatric intensivist, which revealed multiple ill-defined hypoechoic lesions in the spleen (Figures 1, 2) and liver. The child was empirically treated with intravenous (IV) meropenem for presumed melioidosis based on the splenic abscesses seen on the bedside ultrasound and the risk factor of being a local resident in Sarawak, a known endemic area of melioidosis [3].

 

During the patient’s stay in the ICU, he subsequently developed acute respiratory distress syndrome and required high ventilator settings. This was further complicated by pneumothorax and pneumomediastinum requiring chest tube insertion. His condition was further complicated by antibiotic-associated pseudomembranous colitis with bowel ischemia, perforation, and adhesions, for which bowel resection and primary anastomosis was performed.

His blood culture result showed *Burkholderia pseudomallei*, and melioidosis indirect immunofluorescent assay was positive (ELISA IgM [enzyme-linked immunosorbent assay immunoglobulin M] titer 1:1280) confirming the diagnosis of melioidosis. IV meropenem was continued for 10 days and then switched to ceftazidime following the antibiotic sensitivity result. The child recovered after four months of hospitalization and was finally discharged home with normal cognitive and mental function.

## Discussion

This case report highlights the use of POCUS examination in making early diagnosis in patients with a suspicion of melioidosis. Making a diagnosis of melioidosis in children is challenging as the initial presentation may be non-specific. Melioidosis is less common in children compared with adults and has been reported to constitute 5 to 15% of all cases [6]. Moreover, children with melioidosis may present with undifferentiated fever without focal infection. A study in Sarawak, Malaysia, showed that 17% of children with undifferentiated fever had disseminated melioidosis [3]. Ceftriaxone is often the first-line empirical antibiotics for previously healthy patients presenting with septic shock. For this patient, the initial empirical antibiotic coverage would be inadequate if routine care was given.

The intra-abdominal organ most commonly affected by melioidosis is the spleen followed by liver and kidneys. A recent study including 27 Thai children who had confirmed diagnosis by either serology and/or culture reported that children with confirmed melioidosis had a higher proportion of liver and splenic abscess when compared with non-confirmed cases (44% vs. 12%; p < 0.01) [7]. Patients who had liver abscess, splenic abscess, or skin/soft tissue infection were more likely to have melioidosis, with a likelihood ratio of 5.6, 4.0, and 2.2, respectively, and with specificities of 0.94, 0.89, and 0.68, respectively [7]. Without liver abscess, splenic abscess, or soft tissue infection, the negative predictive value of melioidosis was 0.90 [7]. Another study in adults showed that intra-abdominal abscess was common in patients with melioidosis [8]. One or more abscesses were present in the liver and/or spleen in 33% of all participants with melioidosis. Among those with abscess, multiple lesions were noted in 70% of cases with hepatic abscess and 88% of cases with splenic abscess. Moreover, a study in Singapore revealed that 90% of adult patients with splenic abscess had multiple abscesses, which were most commonly caused by disseminated melioidosis (71%) [5].

Abscesses appear on ultrasound as hypoechoic lesions, target lesions, and multiloculated lesions [1]. Splenic and hepatic lesions can be single or multiple. The size is usually small and discrete. A previous study reported that the size of splenic lesion in the CT scan ranges between 0.5 cm and 1.5 cm [9]. Concurrent splenic and liver abscess was highly suggestive of melioidosis (adjusted odds ratio = 11.3; 95% confidence interval = 1.6-77.5; p = 0.014) [1,10].

Cases of melioidosis with liver and/or splenic abscess may not have abdominal signs or symptoms [7]. Abdominal tenderness was reported in only 42%, hepatomegaly for liver abscess in 75%, and splenomegaly for splenic abscess in only 33% of all children with melioidosis [7], together with the study in adults, which reported that intra-abdominal abscesses were cryptic in three-quarters of cases [8].

These results lead to a suggestion that patients with a suspicion of melioidosis such as those who live or come from the endemic area should undergo imaging for early detection of the intra-abdominal abscess even if the patient is asymptomatic or has normal abdominal examination. If the imaging shows splenic abscess with or without liver involvement, it is highly suggestive of melioidosis.

With the advance of POCUS examination in recent years, pediatric emergency physicians and intensivists are now able to perform POCUS in the emergency department (ED) or ICU [11]. Due to its non-invasive nature, radiation-free, and prompt availability comparing with CT scan or ultrasound performed by a radiologist, POCUS examination has now become a part of bedside patient evaluation and therefore potentially results in a decrease in patient morbidity, diagnostic delays, length of stay, and need for the additional imaging. A study of children with suspected appendicitis has demonstrated the ability of POCUS in decreasing ED length of stay when compared with those requiring formal ultrasound (154 and 288 minutes, respectively; p < 0.001) [12].

For pediatric patients, POCUS has been used previously to detect various non-traumatic intra-abdominal pathologies with high diagnostic accuracy, such as appendicitis (sensitivity of 86% and specificity of 91%) [13] and intussusception (sensitivity of 95% and specificity of 99%) [14]. POCUS is also useful in diagnosing some tropical infectious diseases such as the Focused Assessment with Sonography for HIV-associated tuberculosis (FASH) [15]. Application of POCUS to detect splenic and liver abscess will also be helpful in the early diagnosis and management of a difficult-to-diagnose condition such as melioidosis.

## Conclusions

POCUS performed by a trained physician team can be helpful in early provisional diagnosis of melioidosis, which is a less common disease in the pediatric population, by detecting cases of splenic and/or hepatic abscesses in endemic areas. This results in promptly appropriate treatment while waiting for the definitive pathological diagnosis and therefore decreases morbidity and mortality of the patients.
